# A complete morphological characterization of all life stages of the phorid fly *Megaselia scalaris*

**DOI:** 10.1038/s41598-023-50200-6

**Published:** 2023-12-21

**Authors:** Jayakumar Pallavi, Harshita Snehal, Rakshita Sukruth Kolipakala, Daniela Salazar, Mrunal Hanbar, Larina Bejoy Chiramel, Khushi Alok Jha, Sai Bhumica Lakshmi Venkatesh, Tanishka Dayanand Shetty, Navya Madhusudan, Amrutha Mohan, Amulia John, Naomi Deep D’souza, Priyanka Sheet, Deepesh Nagarajan

**Affiliations:** 1 Department of Biotechnology, M.S. Ramaiah University of Applied Sciences, Bangalore, 560054 India; 2 Ecology and Genetics Research Unit, University of Oulu, 90014 Oulu, Finland; 3 Department of Microbiology, St. Xaviers College, Mumbai, 400001 India

**Keywords:** Zoology, Entomology

## Abstract

*Megaselia scalaris*, commonly known as the scuttle fly, is a cosmopolitan species in the family Phoridae. It is an easily cultured fly species that is an emerging model organism in the fields of genetics and developmental biology. Its affinity for carrion and its predictable life cycle makes it useful in the field of forensic science for estimating the post-mortem interval (PMI) of human remains. Cases of human myasis caused by *M. scalaris* have also been reported in the medical literature. Despite its ubiquitous prevalence and its relevance across multiple fields, its morphology has not been adequately characterized. Here, we report the complete morphological characterization of all lifestages of *M. scalaris*, ranging from egg to adult. Scanning electron microscopy has enabled us to uncover morphological features and developmental processes that have previously not been reported in the literature. Our data lays the groundwork for future genetic studies: a morphological characterization of the wild type must be performed before mutants displaying different phenotypes can be identified. In this vein, we also report the observation of a acephalic, or 'headless’, adult phenotype whose study could yield insights into the process of cephalogenesis. Finally, all morphological features observed have been compiled into an ’atlas’ that should be of use to all workers in the field.

## Introduction

*Megaselia scalaris*, commonly known as the “scuttle fly”, is a member of the subfamily Metopininae and the newly reinstated tribe Gymnophorini. The Phoridae family contains over 225 genera and over 2500 species^1^. This species can be distinguished from other phorid flies through its characteristic black stripes on its dorsal abdomen, thick costal vein, and humpbacked appearance^2^. Other distinctive features for this species include a shorter and broader sixth tergite on female adults and a single strong bristle on left side of the epandrium on male adult^3^. The life cycle of *M. scalaris* consists of four stages: eggs, larva, pupa, and adult, with the larval stage consisting of three sequential instars. The life cycle spans 1–3 weeks^4^, and is dependant on temperature and humidity. *M. scalaris* larvae can subsist on a variety of food sources, including carrion^5^, bananas^6^, and even paint^7^. *M. scalaris* breeds prodigiously; more than 350 eggs can be laid by a single female^8^. These traits make *M. scalaris* very easy to rear in the laboratory.

*M. scalaris* has been increasingly used as a model organism in the fields of genetics and developmental biology^8^. Wolf et al. used *M. scalaris* for studying the role of histone acetylation in spermiogenesis^9^, observing that histone acetylation occurs before histone replacement with protamines in spermatid nuclei. *M. scalaris* adults display an unusual style of locomotion for phorid flies: they ’scuttle’ in short bursts followed by periods of rest. Tretyn et al.^10^ believe that understanding the physiological basis for this behaviour could advance the field of neuromuscular physiology. Tretyn et al. also noted differences between the neuromuscular junctional architecture and electrophysiology of *D. melanogaster* and *M. scalaris* larvae. Orgogozo and Schweisguth^11^ noted differences in the peripheral nervous systems (PNS) of *D. melanogaster* and *M. scalaris*. Such differences across phorid and callophoridae flies (blow flies) were used to trace the evolutionary origins of the PNS. Yoo et al.^12^, Smith^13^, and Kwak^14^, independently used *M. scalaris* to study apoptosis.

*M. scalaris* has gained importance in the field of forensic entomology. Determining the post-mortem interval (PMI) of a corpse using developmental data for *M .scalaris* was found to be more accurate than the PMI calculated using callophoridae flies, especially for corpses found indoors and in winter conditions. Reibe and Madea^15^ demonstrated this using three corpses autopsied in Germany. Greenberg and Wells^16^ described the isolation of *M .scalaris* from two corpses in Chicago, and provided temperature-dependant developmental data for *M. scalaris* larvae .

*M. scalaris* is a clinically relevant organism. It has been reported as the etiological agent in several clinical cases of myasis, such as: urinary myasis (Egypt^17^) , urinogenital myasis (India^18^, Iran^19^), intestinal myasis (Egypt^20^), nasopharyngeal and wound myasis (Kuwait^21^).

Given its importance in basic research, forensics, and medicine, the existing literature describing the morphological and ultrastructural characterization of *M. scalaris* remains fragmentary and incomplete. Disney^2^ published an influential treatise dealing with the morphology, ecology, development and identification of phorid flies. Due to the large scope of this work, covering several different species in the family Phoridae, and due its reliance on hand-drawn diagrams rather than micrographs, it cannot be used as (and was not designed as) a comprehensive resource dealing with *M. scalaris* morphology.

Other workers have used electron microscopy to characterize different stages of the *M. scalaris* life cycle. However, this characterization is fragmentary as the work was performed by different teams, using different instruments, using different strains of *M. scalaris strains*, and in different countries, making straightforward comparisons between the data difficult. The egg life stage was characterized by Furukawa et al.^22^ in Japan, Greenberg and Wells^16^ in Chicago, and Wolf *et. al*^23^ using the “Wienn” strain. The larval life stages were characterized by Boonchu et al.^24^ and Sukontason et al.^25^ in Thailand, Ismail^26^, Shaheen et al.^27^, and Mayzad et al.^28^ in Egypt, and Machkour-M’Rabet et al.^29^ in Mexico. The pupal lifestage was characterized by Sukontason et al.^30^ in Thailand and Braga et al.^31^ in Brazil. Furthermore, the individual reports are sometimes contradictory to each other and to our current work. We have addressed these contradictions in the results section.

In this work, we have used light microscopy (LM) and environmental scanning electron microscopy (ESEM) as complementary tools while characterizing *M. scalaris*. Light microsocopy can reveal pigmentation patterns that ESEM cannot detect. ESEM can reveal detailed surface ultrastructures that light microscopy cannot detect, due to both the limited resolution of photons and the optically translucent nature of our samples. Using these tools, we have characterized the egg, larval, pupal, and adult live stages of *M. scalaris* in detail. Our observations fill in the missing gaps and correct previous errors in the existing literature. While the main body of this article reports novel structural and developmental features of *M. scalaris*, we have also provided an ’atlas’ describing all observed morphological features in the supplementary material (Supplementary Text S1) which should be useful to all workers in the field. Furthermore, we report the observation of an acephalic, or ’headless’, adult phenotype that was previously not described in the literature. The further study of this mutant could yield insights into the process of cephalogenesis.

## Results and discussion

*M. scalaris* was isolated in Mumbai, India. The specimen’s identity was confirmed via mitochondrial 16s rRNA sequencing, performed on undifferentiated larvae by Sakhala Enterprises (Bangalore). The 16s rRNA sequences have been deposited in Genbank (forward: OR121062, reverse: OR121063). All previously unreported morphological features and developmental processes with of the egg, first instar larval, second instar larval, third instar larval, pupal, and adult (male and female) lifestages are described below.

Morphological features that we observed but were previously reported, along with our newly observed morphological and developmental features, have been made available as an ’atlas’ in the supplementary material (Supplementary Text S1).

### The egg lifestage

**Figure 1:**
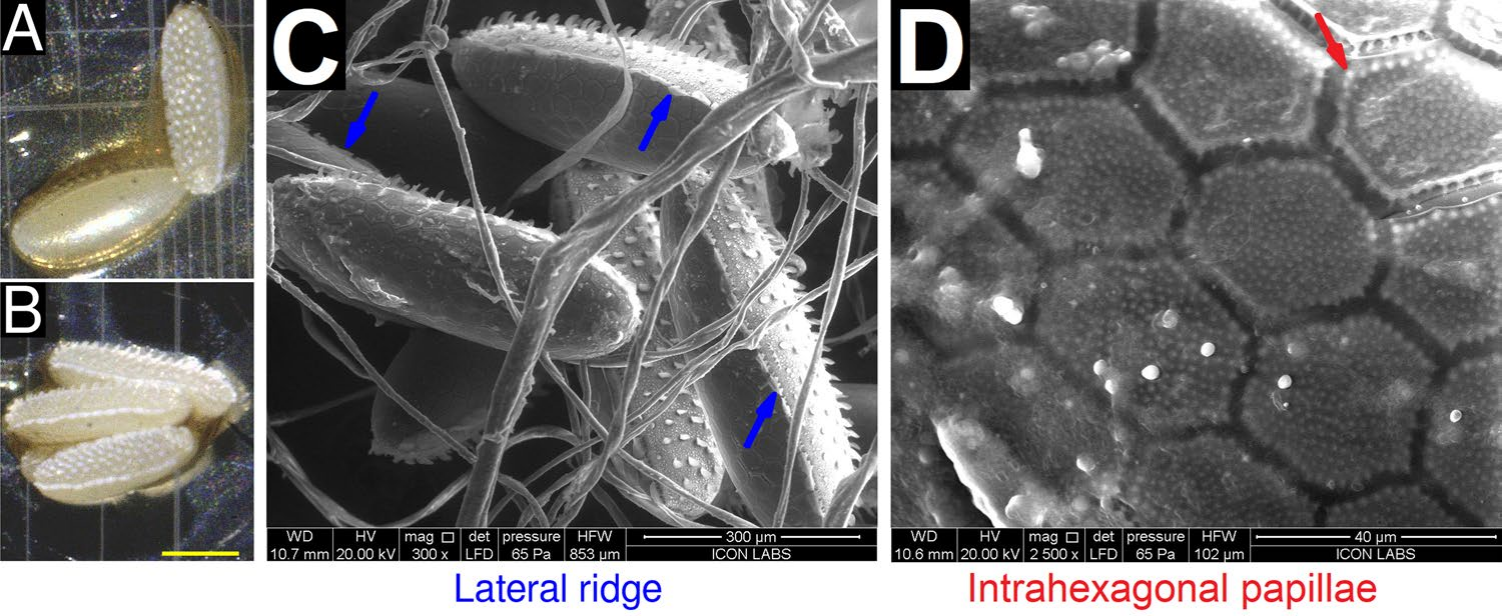
Morphological characterization of *M. scalaris* eggs using light and electron microscopy, (**A**) Dorsal (above, right) and ventral (below, left) views of eggs under light microscopy at 40$\times$ magnification. The dorsal surface appears rough due to dorsal spicula. The ventral surface appears smooth. (**B**) Lateral view of eggs. Dorsal spicula are clearly visualized. A lateral ridge separating the dorsal and ventral surfaces is visible, but is better visualized using electron microscopy. Scale bar (yellow) represents 0.25 mm. (**C**) Lateral view of multiple eggs at 300$\times$ magnification. The lateral skirt (blue arrow) is visible on three separate eggs. (**D**) Ventral view at 2500$\times$ magnification. Papillae within each hexagonal tile (interhexagonal papillae, red arrow) are clearly visible.

The eggs of *M. scalaris* are small, ovoid, and yellow–white (Fig. 1A,B). The dorsal surface appears rough, possessing spicula of three different sizes: $\alpha$-spicula, $\beta$-spicula, and $\gamma$-spicula (Supplementary Text S1). In contrast, the ventral surface appears smooth (Fig. 1A). The dorsal and ventral surfaces are separated by a lateral ridge (Figure 1C). This ridge is composed of thin rectangular segments. The smooth ventral surface is composed of hexagonal tiles (Fig. 1D). Here, we report the presence of small protuberances on the surface of the ventral hexagonal tiles that we term as intrahexagonal papillae.

We hypothesize that the dorsal $\alpha$-spicula may help the egg attach to fibrous surfaces via mechanical interlocking. The $\beta$- and $\gamma$-spicula may also serve some role in adherence to a substrate. Further, we hypothesize that the papillae on the smooth ventral surface may help attach the egg to hard, smooth surfaces. However, further experiments are required to validate these hypotheses.

Our observations are mostly in agreement with work published by Furukawa et al.^22^ on *M. scalaris* specimens isolated in Japan. However, our work offers clearer images acquired at higher resolutions, allowing us to visualize the previously unreported intrahexagonal papillae. Similar work was performed by Greenberg and Wells on specimens isolated from a corpse in Chicago, USA^16^. In this case, the eggs displayed extensive structural deformation, possibly occurring during fixation prior to SEM. As we performed ESEM (environmental SEM), our specimens required no treatment prior to visualization.

### The larval instars

**Figure 2:**
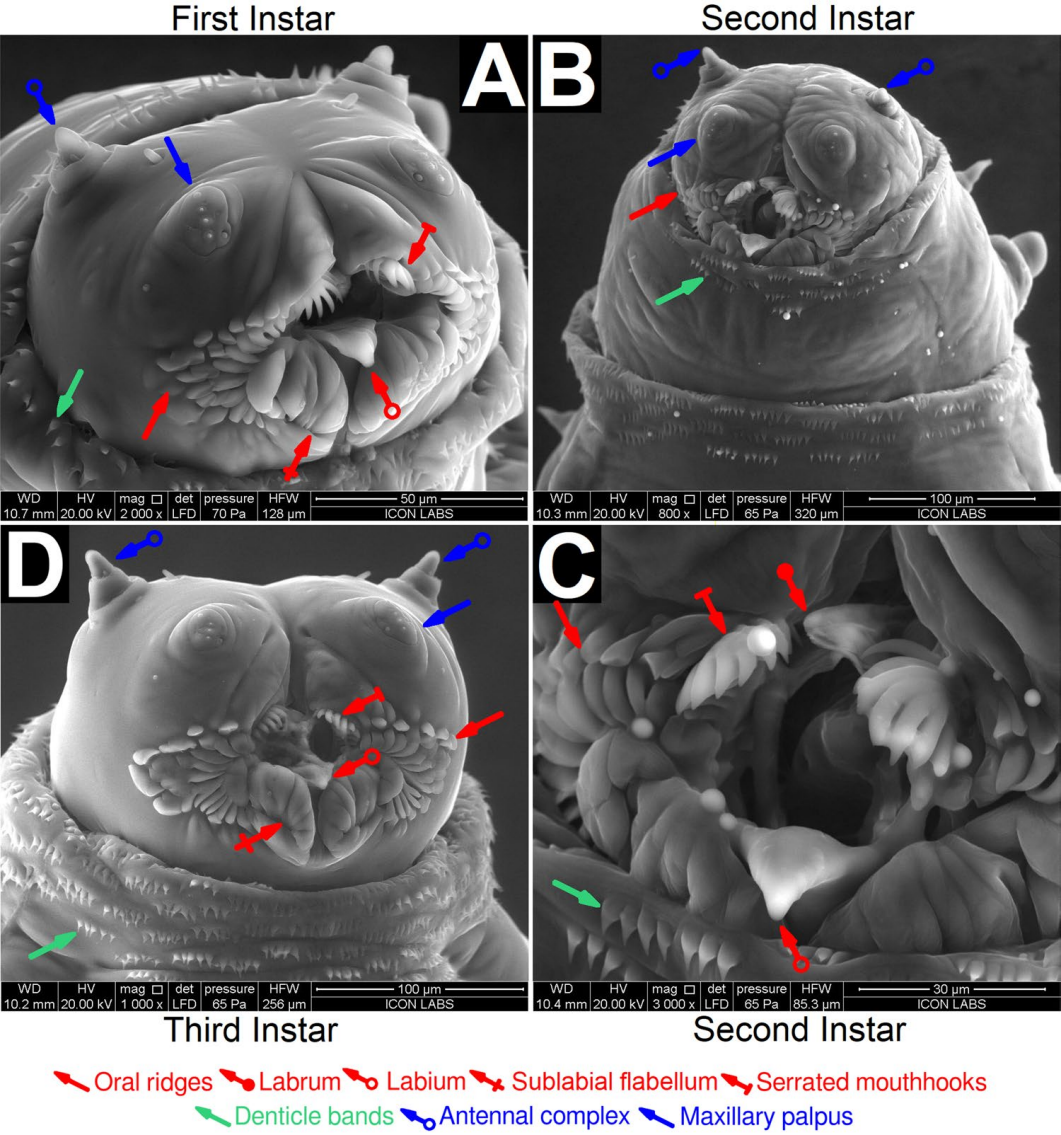
ESEM characterization of *M. scalaris* larval antero-ventral cephala. (**A**) First instar larva at 2000$\times$ magnification. (**B**) Second instar larva at 800$\times$ magnification. (**C**) Second instar larval oral cavity at 3000$\times$ magnification. (**D**) magnification. The oral cavity consists of the labium (red arrows, hollow end), serrated mouthhooks (red arrows, flat end), and sublabial flabellum (red arrows, crossed end), surrounded by oral ridges (red arrows) for all instars. The labrum (red arrow, round end) was only clearly observed for second instar larvae. All larval cephala also possess the maxillary palpus (blue arrows) and the antennal complex (blue arrows, hollow end), and are surrounded by denticle bands (green arrows) originating from the first thoracic segment.

The larval lifestage of *M. scalaris* can be subdivided into three instars that can be differentiated based on their size and the presence/absence of pigmentation (Supplementary Text S1). The morphology of the larval cephala (Fig. 2A–D) remains consistent across all instars. An anterio-ventral view reveals cephala possessing an antennal complex, maxillary palpus, and a well developed mouth. The mouth in all instars possesses a labium, a pair of deeply serrated mouthhooks, and a pair of structures we term as the sublabial flabella. The labrum was only observed in second instar larvae, being either absent or recessed into the oral cavity for first and third instar larvae. The mouth is surrounded by oral ridges.

**Figure 3:**
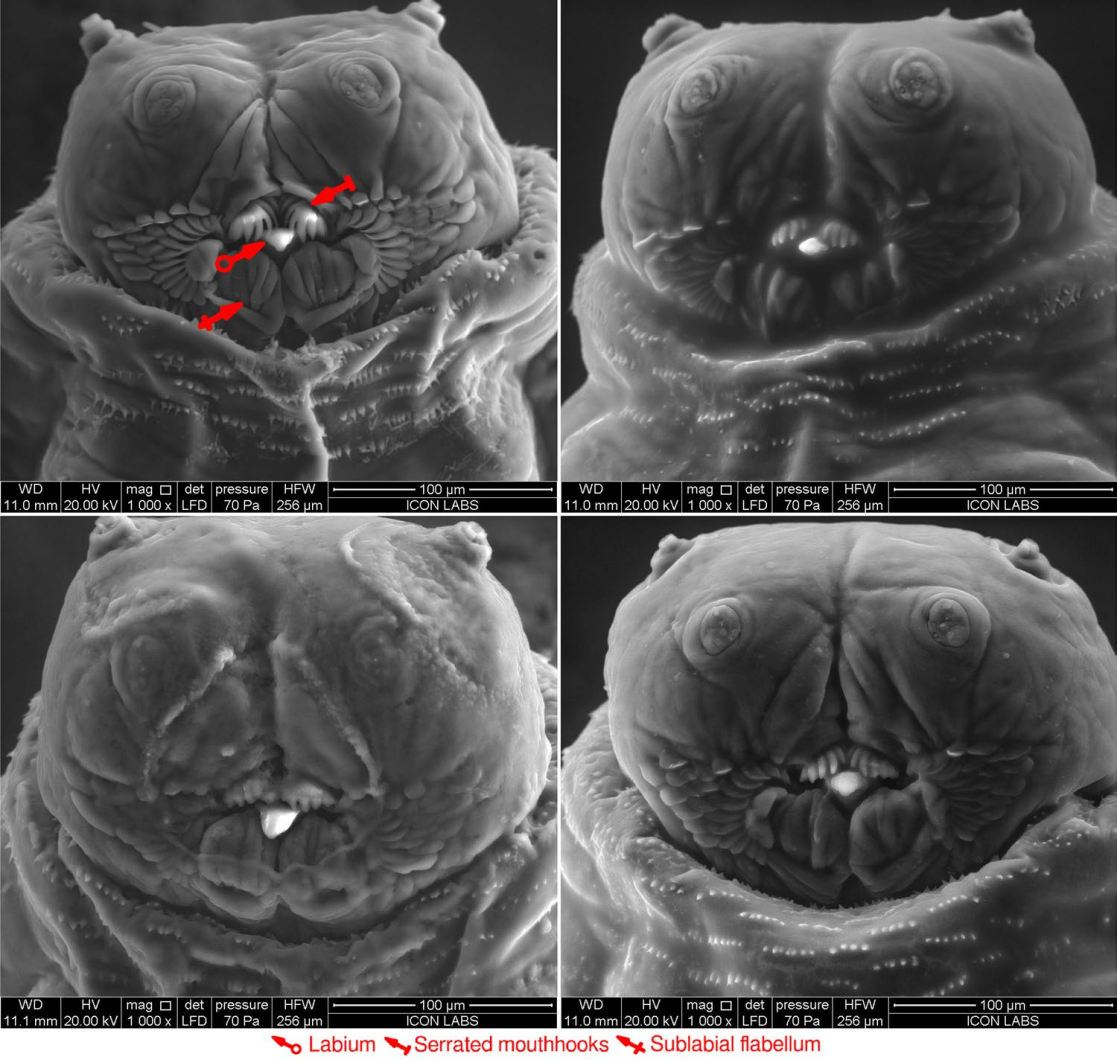
ESEM characterization of the cephalons of four specimens of *M. scalaris* third instar larvae. The labium (red arrow, hollow end), serrated mouthhooks (red arrow, flat end), and sublabial flabellum (red arrow, crossed end) are highlighted for one specimen.

In (Fig. 2D), the third instar larval oral cavity appears open. In Fig. 3, all specimens appear to have closed oral cavities. We believe the serrated mouthhooks, the labium, and sublabial flabella act analogously to human lips, opening and closing the oral cavity whenever necessary.

Every third instar larval cephalon observed possessed a unique pattern of oral ridges (Figs. 2D, 3), akin to fingerprints. Our future research will aim to investigate whether the distinct patterns of oral ridges in third instar larvae can be employed as a tool for phylogenetic analysis. We hypothesize that closely related larvae, such as those belonging to the same *M. scalaris* population, will exhibit similar oral ridge patterns, while those from more distant populations will display differing patterns.

There is some disagreement in the literature regarding the morphology of the first instar larval cephalon: Boonchu et al. did not report the presence of maxillary palpus on the cephalon of first instar larvae isolated in Thailand^24^. These structures were possibly destroyed during the extensive glutaraldehyde fixation process required to prepare larvae for SEM. As we performed ESEM (environmental SEM), our specimens required minimal treatment and were able to retain fine structures.. Sukontason et al.^25^, Ismail^26^, Shaheen et al.^27^, and Machkour-M’Rabet et al.^29^ have characterized third instar larvae isolated in Thailand, Egypt, Egypt, and Mexico, respectively. However, we have captured the larval cephalon in greater detail while also annotating previously unreported morphological features.

### The pupal life stage

The pupal lifestage of *M. scalaris* can be divided into two distinct sub-stages: the early pupal stage, before the emergence of respiratory horns and the development of the carapace. The later pupal lifestage occurs after these events.

**Figure 4:**
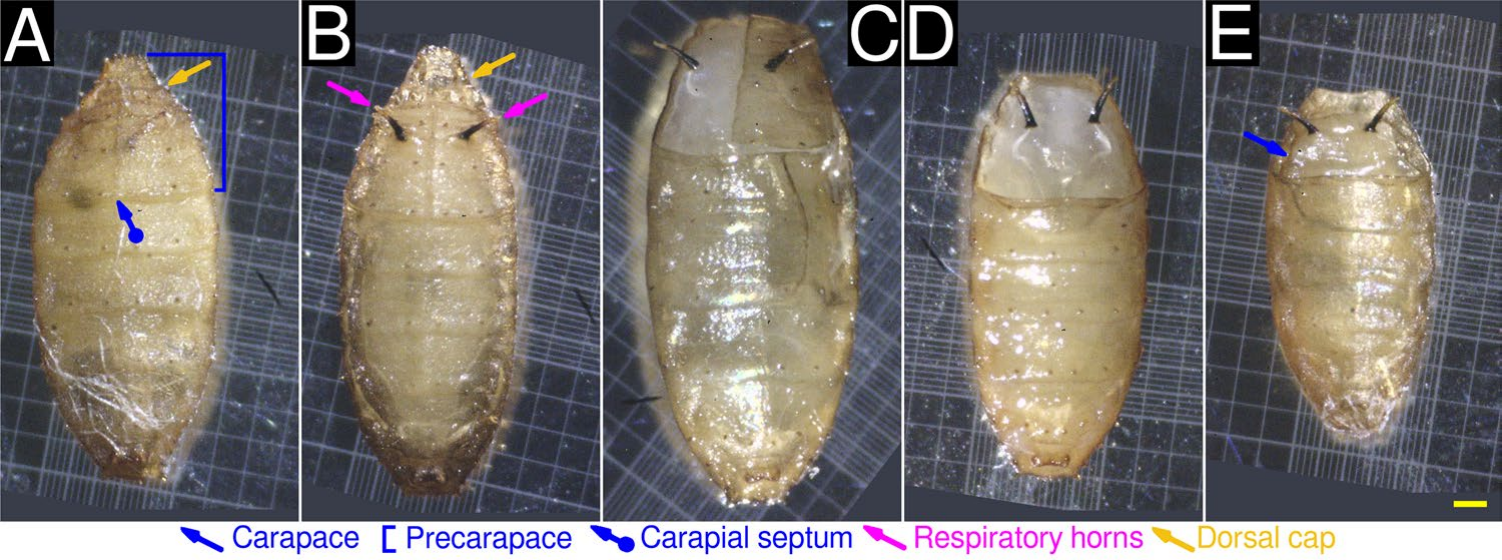
Light microscopic characterization of *M. scalaris* pupae at 20$\times$ magnification. (**A**) Dorsal view of a pupa (early stage) displaying the dorsal cap (yellow arrow), precarapace (blue bracket) and carapogenic septum (blue arrow, round end). The respiratory horns are absent in this stage. (**B**) Development of respiratory horns (magenta arrows). (**C**) Ecdysis of one lateral segment of the precarapace along the carapogenic septum. Also note the ecdysis of the dorsal cap. (**D**) Ecdysis of both lateral segments of the precarapace along the carapogenic septum. (**E**) Pupa (late stage) with fully formed carapace (blue arrow). It should be noted that a different specimen is present on each panel. Background regions not included in the field of view are colored dark gray. For all panels, the scale bar (yellow) represents 0.25 mm.

**Figure 5:**
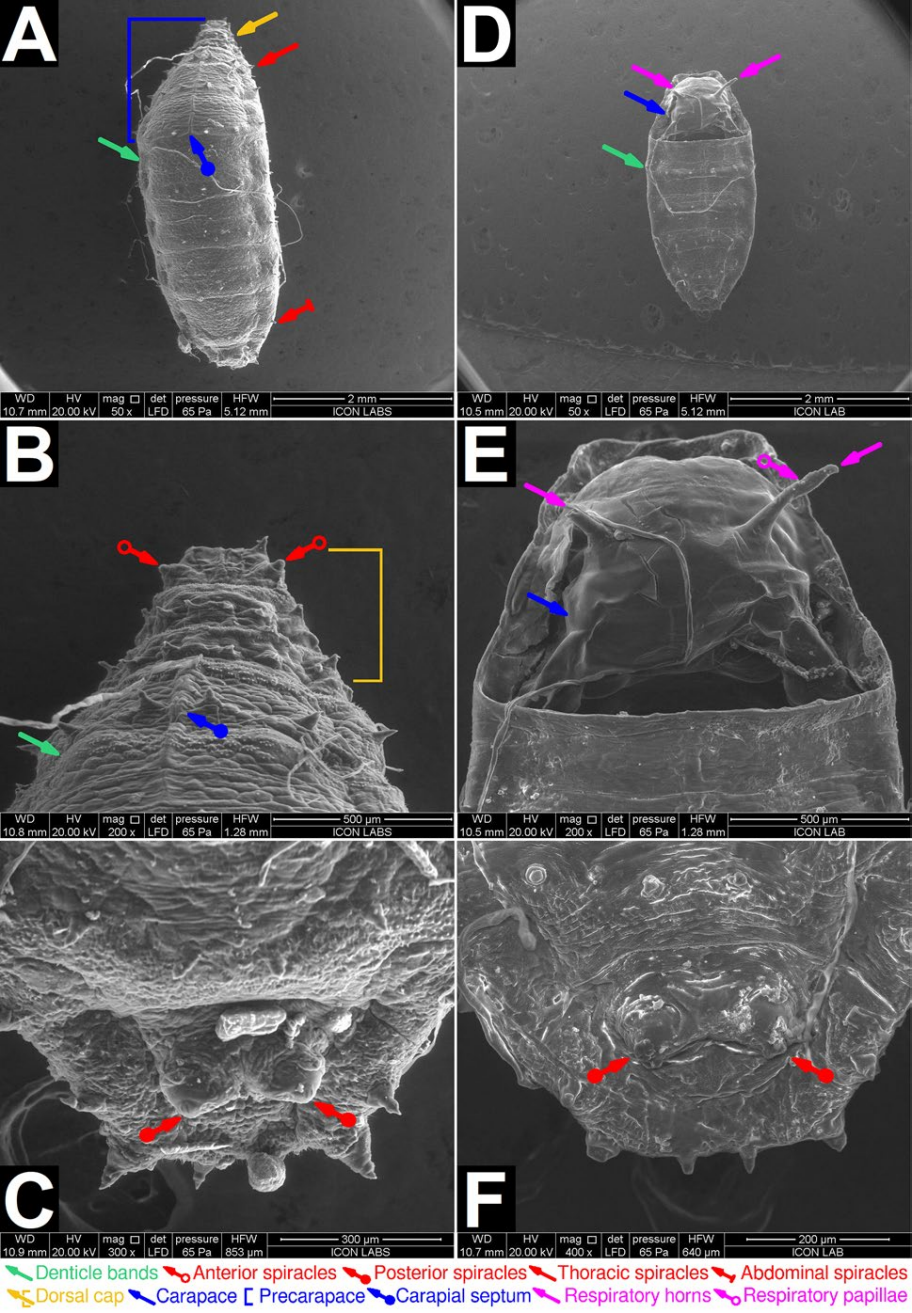
ESEM characterization of *M. scalaris* pupae. ESEM characterization of *M. scalaris* pupae. (**A**) magnification, displaying the dorsal cap (yellow arrow), precarapace (blue bracket), dentice bands (green arrows), thoracic spiracles (red arrow), and abdominal spiracles (red arrow, flat end). The respiratory horns are absent in this stage. (**B**) Pupa (early stage): anterior view (dorsal) at 200$\times$ magnification, displaying the dorsal cap (yellow bracket) and anterior spiracles (red arrows, hollow end). Spiracluar openings (slits) can be seen. (**C**) Pupa (early stage): posterior view (dorsal) at 300$\times$ magnification. Posterior spiracles (red arrows, round end) can be seen, each possessing two spiracular openings (slits). (**D**) Dorsal view of a pupa (late stage) displaying prominent respiratory horns (magenta arrows). The carapace (blue arrow), formed by the fusion of thoracic and abdominal segments, can be seen. (**E**) Pupa (late stage): Anterior view (dorsal) at 200$\times$ magnification. The carapace (blue arrow) is in clear view. Two respiratory horns (magenta arrows) are seen, however it should be noted that the left respiratory horn was damaged. Spirally arranged respiratory papillae (magenta arrow, hollow end) are seen. Respiratory papillae were visualized in greater detail by Sukontason et al.^30^. (**F**) Pupa (late stage): posterior view (dorsal) at 400$\times$ magnification. The posterior spiracles appear to have atrophied. Spiracular openings (slits) were not observed.

The early pupal stage is characterized by a cylindrical, segmented puparium following the same thoracic and abdominal segmentation pattern as third instar larvae (Figs. 4A, 5A). We term the three thoracic segments and three abdominal segments present in the dorso-anterior region as the precarapace (Figs. 4A, 5A). A thin septum bisecting the precarapace can be observed. We term this structure as the carapogenic septum (Figs. 4A, 5A). The anterior end of the precarapace contains the dorsal cap. The pupa retains its larval spiracular network, which includes the thoracic/abdominal (Figure 5A), anterior (Figure 5B), and posterior spiracles (Fig. 5C). The posterior spiracles possess two straight slits. Denticle bands are retained from the larval lifestage.

Pupae in the early stage transition to the late stage through a process we term carapogenesis. Firstly, two respiratory horns emerge on the second abdominal segment of early pupae (Fig. 4B). Secondly, the two lateral segments of the precarapace undergo sequential ecdysis along the carapogenic septum, exposing the underlying soft tissue (Fig. 4C,D). The respiratory horns remain unaffected. The loss of the dorsal cap occurs simultaneously. Thirdly, a chitinous carapace is synthesized and encapsulates the previously exposed soft tissue (Figs. 4E,5D,E).

The later pupal lifestage occurs after the formation of the respiratory horns and carapace. Spirally arranged respiratory papillae can be seen on the surface of the respiratory horns (Fig. 5E). The formation of respiratory horns may be required to compensate for the atrophy of all other spiracles (Fig. 5F). The pupa now displays dorso-ventral flattening. The ventral view of the later pupal lifestage is unremarkable (Fig. 5G,H,I). These pupal lifestages have not been previously reported in the literature.

An intermediate pupal lifestage of *M. scalaris* has been inadvertently characterized using SEM by Sukontason et al.^30^, is mostly in agreement with our observations. Sukontason et al. reported pupae possessing respiratory horns but no carapace, similar to the pupa shown in Fig. 4B. Abdel-Gawad^32^ characterized late stage *M. scalaris* pupae, and is in agreement with our observations. Braga et al.^31^ characterized late stage *M. scalaris* pupae in mummified human remains in Brazil. The pupal specimen presented possessed a carapace but did not possess respiratory horns. Respiratory horns are fragile and may not preserve easily. It should be noted that no other group reported the identification of distinct sub-stages in pupal development.

### The adult lifestage

*M. scalaris* adults exhibit sexual dimorphism. The female is larger than the male^33^. The sexes can also be distinguished by their mouthparts and genitalia. Both sexes are morphologically similar in all other respects. The *M. scalaris* adult body can be divided into the head, thorax, and abdomen. A complete morphological description of adult male and female flies is provided in Supplementary Text S1. Novel morphological features are discussed below.

**Figure 6:**
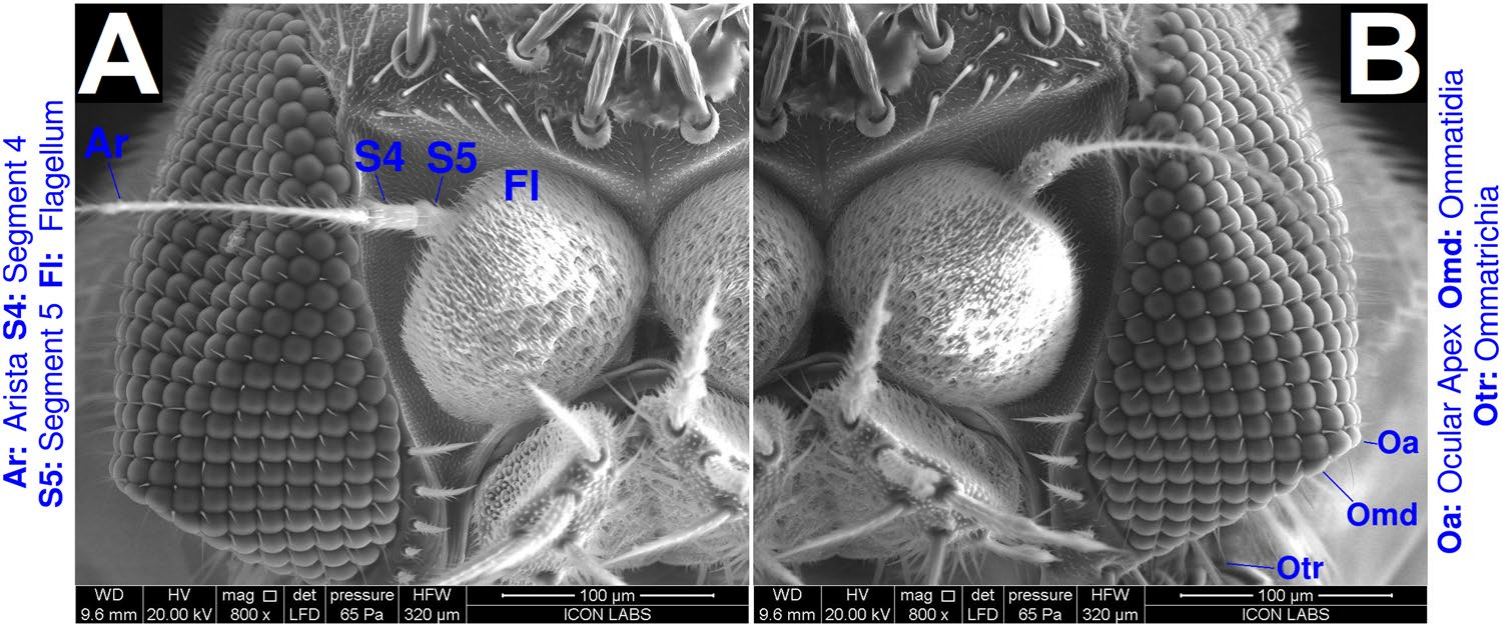
ESEM characterization of adult male *M. scalaris* eyes. (**A,B**) Left and right views of the cephalon respectively, at 800$\times$ magnification, focusing on the eyes and antennae. The antennal complex consists of the distal arista (Ar), followed by segments 4 and 5 (S4,S5) terminating at the flagellum (Fl). The eyes are composed of numerous ommatidia (Omd) with ommatrichia (otr) present at every interstitial space. The ocular apex (Oa) is also highlighted. Note the hexagonal packing of the ommatidia.

The defining feature of the head is the large compound eyes characteristic of all phorid flies. Each compound eye contains hemispherical ommatidia arranged in hexagonal or square close packing. Ommatidia closer to the periphery display hexagonal close packing, while ommatidia closer to the center display square close packing. Ommatrichia are present in every interstitial space between ommatidia. Double ommatrichia are occasionally present. Both our male and female specimens’ eyes deviated from the conventional spherical shape. Their eyes possessed a ventral, pointed structure we term as the occular apex (Fig. 6A,B). It should be noted that the ocular apex has not been reported for *D. melanogaster*^34,35^ or any other scuttle fly species^2^. Interestingly, the ocular apex has not been reported for other strains of *M. scalaris* as well. Sukontason et al. reported the ultrastructure of eyes from *M. scalaris* specimes isolated in Thailand^36^. Their specimens did not possess ocular apexes. The possibility that the ocular apex is an electron microscopic artifact can be eliminated since both eyes on our male specimen, as well as both eyes on our female specimen (Supplementary Text S1), possess ocular apexes. The possibility that such a structure independently arose 4 times by chance is very remote. This leads us to conclude that *M. scalaris* strains possessing an ocular apex may be geographically restricted within a range that may span anywhere from the Mumbai metropolitan area to the Indian subcontinent. Further work is needed to confirm the range of this phenotype.

**Figure 7:**
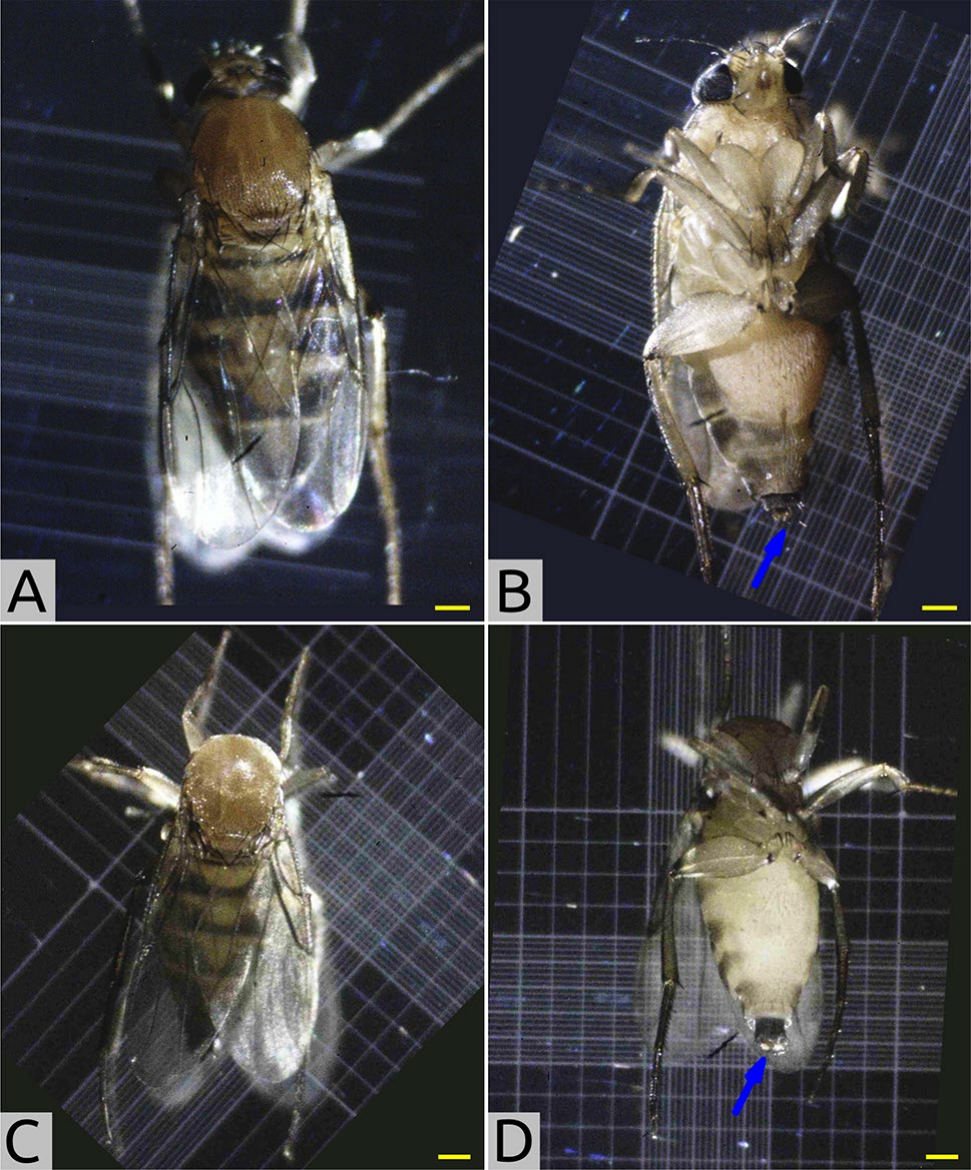
Light microscopic characterization of adult female *M. scalaris* acephalic phenotype: (**A**) Female, dorsal view. Note the characteristic black stripes on every segment of the dorsal abdomen. (**B**) Female, ventral view. The ovipositor is visible at the posterior end (blue arrow). (**C,D**) Acephalic adult female. Background regions not included in the field of view are colored dark blue. For all panels, the scale bar (yellow) represents 0.25 mm.

During the process of image collection via light microscopy, we noticed an acephalic, or ’headless’, phenotype (Fig. 7C,D). Figures 7A,B show adult female flies for reference. 4 acephalic flies were observed in a cohort of 100 photographed flies, indicating that the frequency of occurrence of this phenotype is 4%. We speculate that the head may be damaged during the process of carapogenesis, during which the precarapace surrounding the pupal cephalon is ecdysed before the formation of the carapace. The pupal head is exposed and vulnerable to mechanical damage during this process. Further study is warranted to explore the genetic and developmental processes that lead to this phenotype.

In conclusion, we have morphologically characterized all life stages of *M. scalaris*. While we have only discussed novel morphological and developmental features in this work, we have made available an atlas containing the organism’s complete morphological characterization in Supplementary Text S1. We hope this work and the atlas will be useful to the community.

## Methods

### Isolating *M. scalaris*

*M. scalaris* specimens were isolated from Malabar Hill, Mumbai, India (pin code: 400006). Flies were isolated using Whiskas catfood (adult, chicken in gravy). A pouch of catfood was placed in an exposed bowl in the area mentioned. Larvae were collected and used for subsequent experiments. This culture is currently maintained in our laboratory. Workers interested in acquiring our culture can contact the corresponding author.

### Culturing *M. scalaris*

Flies were subcultured using catfood media of the following composition: 75% catfood (homogenized using a mortar and pestle), 24% distilled water, 1% agar. Vials were incubated at room temperature. This media was autoclaved and 10 mL aliquots were poured into standard fruitfly vials. Culturing *M. scalaris* requires meat, and catfood was the most convenient source because: (1) catfood is sold in small packs of 85g, making it convenient for preparing small batches of vials; (2) unopened catfood can be stored at room temperature without the need for refrigeration; (3) catfood is processed into small, chewable cubes, minimizing the need for extensive homogenization; and (4) the composition of catfood from the same manufacturer will remain constant over different batches.

### Light microscopy

A trinocular stereo microscope with an attached camera (SHSM-1007 TDLED, Ski-Hi Optical Instruments) was used for this study. All specimens were placed on a hemocytometer for scale. All images were acquired at 20–40$\times$ magnification.

### Scanning electron microscopy

Environmental scanning electron micoscopy (ESEM) was performed using the FEI Quanta 200 Scanning electron microscope at Icon Labs Pvt. Ltd., Mumbai. Deceased larvae were used for ESEM after treatment with 70% isopropanol. Deceased adults were used for ESEM after exposure to a lethal dose of chloroform. Live eggs were used for ESEM. No fixation was necessary prior to performing ESEM.

### Supplementary Information

Supplementary Information.

## Data Availability

The *M. scalaris* mitochondrial 16s rRNA sequences generated during this study have been deposited in Genbank (forward sequence accession number: OR121062, reverse sequence accession number: OR121063).
